# Extreme-Phenotype Genome-Wide Association Analysis for Growth Traits in Spotted Sea Bass (*Lateolabrax maculatus*) Using Whole-Genome Resequencing

**DOI:** 10.3390/ani14202995

**Published:** 2024-10-17

**Authors:** Zhaolong Zhou, Guangming Shao, Yibo Shen, Fengjiao He, Xiaomei Tu, Jiawen Ji, Jingqun Ao, Xinhua Chen

**Affiliations:** 1 Fuzhou Institute of Oceanography, State Key Laboratory of Mariculture Breeding, Key Laboratory of Marine Biotechnology of Fujian Province, College of Marine Sciences, Fujian Agriculture and Forestry University, Fuzhou 350002, China; 5220714019@fafu.edu.cn (Z.Z.); shaoheian@163.com (G.S.); yiposhen512@gmail.com (Y.S.); 1220791011@fafu.edu.cn (F.H.); 52325014043@fafu.edu.cn (X.T.); 52325014009@fafu.edu.cn (J.J.); 2 Southern Marine Science and Engineering Guangdong Laboratory, Zhuhai 519000, China

**Keywords:** *Lateolabrax maculatus*, whole genome resequencing, genome-wide association analysis, growth traits

## Abstract

**Simple Summary:**

Fish farming provides an efficient means of obtaining high-quality protein for humans. However, the decreased growth rate of aquaculture fish due to germplasm degradation often increases production costs and reduces economic benefits. Therefore, it is particularly important to develop fast-growing varieties and elucidate the genetic mechanisms underlying growth traits. In this study, we identified 50 growth-related markers in the genome of *Lateolabrax maculatus*, an important marine aquaculture species, with the phenotypic variance explained up to 15.82%, which will help in marker-assisted breeding for fast-growing varieties. Additionally, 47 growth-related candidate genes were annotated, and the functions of some of these genes in growth traits have been confirmed in mice or zebrafish through gene knockout or knockdown experiments. The 47 candidate genes are mainly associated with the metabolism of energy, glucose, and lipids and the development of musculoskeletal and nervous systems, which may act as drivers for the growth of *L. maculatus*. Altogether, our study identified growth-related markers and candidate genes, which will help develop the fast-growing varieties of *L. maculatus* and elucidate genetic mechanisms underlying its growth traits.

**Abstract:**

Spotted sea bass (*Lateolabrax maculatus*) is an important marine economic fish in China, ranking third in annual production among marine fish. However, a declined growth rate caused by germplasm degradation has severely increased production costs and reduced economic benefits. There is an urgent need to develop the fast-growing varieties of *L. maculatus* and elucidate the genetic mechanisms underlying growth traits. Here, whole-genome resequencing technology combined with extreme phenotype genome-wide association analysis (XP-GWAS) was used to identify candidate markers and genes associated with growth traits in *L. maculatus*. Two groups of *L. maculatus*, consisting of 100 fast-growing and 100 slow-growing individuals with significant differences in body weight, body length, and carcass weight, underwent whole-genome resequencing. A total of 4,528,936 high-quality single nucleotide polymorphisms (SNPs) were used for XP-GWAS. These SNPs were evenly distributed across all chromosomes without large gaps, and the average distance between SNPs was only 175.8 bp. XP-GWAS based on the Bayesian-information and Linkage-disequilibrium Iteratively Nested Keyway (Blink) and Fixed and random model Circulating Probability Unification (FarmCPU) identified 50 growth-related markers, of which 17 were related to body length, 19 to body weight, and 23 to carcass weight. The highest phenotypic variance explained (*PVE*) reached 15.82%. Furthermore, significant differences were observed in body weight, body length, and carcass weight among individuals with different genotypes. For example, there were highly significant differences in body weight among individuals with different genotypes for four SNPs located on chromosome 16: chr16:13133726, chr16:13209537, chr16:14468078, and chr16:18537358. Additionally, 47 growth-associated genes were annotated. These genes are mainly related to the metabolism of energy, glucose, and lipids and the development of musculoskeletal and nervous systems, which may regulate the growth of *L. maculatus*. Our study identified growth-related markers and candidate genes, which will help to develop the fast-growing varieties of *L. maculatus* through marker-assisted breeding and elucidate the genetic mechanisms underlying the growth traits.

## 1. Introduction

The spotted sea bass (*Lateolabrax maculatus*) is an important marine economic fish in China, widely distributed along the coasts of China, extending to the southernmost region of the Indo-China Peninsula and reaching as far north as the Bohai Gulf [1]. Large-scale farming operations were launched following the breakthroughs in the artificial breeding technology for *L. maculatus*. In 2023, the annual production of *L. maculatus* reached 247,000 t in China, ranking third among maricultured fish and representing a 13.24% increase compared to 2022 [2]. However, the decreased growth rate caused by germplasm degradation has severely restricted the sustainable development of the sea bass industry [3]. Therefore, it is of great significance to breed fast-growing varieties and uncover the genetic mechanisms underlying growth for the sustainable development of the *L. maculatus* industry.

Growth is one of the most valuable traits, as it exerts a direct influence on production and economic performance [4,5]. Many studies have suggested that growth is a polygenic trait influenced by genes in multiple physiological pathways, such as those regulating metabolism, muscle growth, bone formation, and appetite. For example, genes on the endocrine somatotropic axis, including growth hormone (*GH*), growth hormone receptor (*GHR*), insulin-like growth factors (*IGFs*), growth hormone-releasing hormone (*GHRH*), growth hormone-inhibiting hormone (*GHIH*), and pituitary adenylate cyclase-activating polypeptide (*PACAP*) are thought to have a strong influence on growth [6,7,8,9]. These genes typically exert both somatic and metabolic effects. Additionally, genes regulating muscle growth, such as myostatin (*MSTN*), myogenic regulatory factors (*MRFs*), myomaker (*MYMK*), and myomixer, have been proven to be essential for muscle development. Notably, *MSTN* has been used to improve the genetic growth of fish. *MSTN* knockout has been shown to contribute to increased muscle growth in Olive Flounder (*Paralichthys olivaceus*) [10], channel catfish (*Ictalurus punctatus*) [11], common carp (*Cyprinus carpio*) [12], and red sea bream (*Pagrus major*) [13]. Besides the genes on the endocrine somatotropic axis and those regulating muscle growth, parvalbumin genes, which play an important role in muscle relaxation, have been suggested to be associated with growth traits in Asian seabass (*Lates calcarifer*) [14]. *MC4R*, a gene involved in appetite and energy homeostasis, is also implicated in the regulation of growth [15]. However, the growth-related genes and regulatory pathways are only partially understood, and the genetic mechanisms underlying growth remain largely elusive. Identifying the regulatory genes and pathways related to growth will not only lay the foundation for understanding the genetic mechanisms of growth-related traits but also provide target genes for gene editing or marker-assisted selection breeding.

Genome-wide association analysis (GWAS) is a powerful approach to locating the loci associated with the target traits by analyzing the correlation between genetic markers across the entire genome and target traits. The markers for traits of interest can be used for marker-assisted breeding, which is of great value for species with a long growth period, including *L. maculatus* (approximately four years) since traditional breeding methods such as mass selection are often time-consuming. Additionally, GWAS is applied to identify novel candidate genes and reveal the genetic mechanism for a particular phenotype, including growth-related traits. At present, GWAS for growth-related traits has been carried out in several aquaculture fishes, including *L. maculatus* [3], large yellow croaker (*Larimichthys crocea*) [5,16], rainbow trout (*Oncorhynchus mykiss*) [17], Atlantic salmon (*Salmo salar*) [18,19], common carp (*Cyprinus carpio*) [20]. Recently, Zhang et al. [3] conducted the first genome-wide association study for growth traits using whole-genome resequencing and detected 66 growth-related SNPs based on a Mixed Linear Model (MLM) [21] and 3.75 million SNPs, which laid the basis for further elucidation of genetic mechanisms underlying growth traits.

Extreme-phenotype genome-wide association study (XP-GWAS) is an improved GWAS, which can effectively reduce the sample size required [22]. In this study, we sequenced two groups of *L. maculatus*, consisting of 100 fast-growing and 100 slow-growing individuals with significant differences in body weight (BW), body length (BL), and carcass weight (CW) traits, and obtained 19.50 million SNPs, of which 4.53 million SNPs were used for subsequent XP-GWAS. We also compared the performance of the MLM, Bayesian-information and Linkage-disequilibrium Iteratively Nested Keyway (Blink) [23] and Fixed and random model Circulating Probability Unification (FarmCPU) [24] in accurately determining SNPs, found that except for MLM, both Blink and FarmCPU could effectively reduce false positives and false negatives. We identified 50 growth-related markers, with the highest *PVE* of these markers reaching 15.82%. We also annotated 47 growth-associated candidate genes. The functions of some of these genes in growth have been confirmed in mice or zebrafish through gene knockout or knockdown experiments, while some were newly identified as growth-related genes. We discussed their role in growth. Our study not only identified the markers of significant breeding value but also provided new insights into the mechanisms underlying growth traits.

## 2. Materials and Methods

### 2.1. Materials

The *L. maculatus* used in this study were provided by Fujian Minwei Industrial Co., Ltd. (Fuding city, China). To exclude the influence of age on growth differences, all individuals were selected from the offspring of the same breeding population, which consisted of 100 randomly selected broodstock with a random mating design. All the offspring were raised in marine cages under identical conditions to eliminate the influence of factors like density or food availability on growth differences. The number of offspring was estimated to be 6 million, and their weight was approximately 1202.42 ± 31.82 g (mean ± standard deviation (SD)) based on the results of a random sampling of 50 individuals. We selected 200 individuals from the offspring, consisting of two groups with obvious differences in growth rate. Specifically, individuals in the fast-growing group weighed over 1361.52 g (mean + 5 SDs), while those in the slow-growing group weighed less than 1043.32 g (mean − 5 SDs). Each group contained 100 individuals. In addition to body weight, we also measured body length (BL, length from the beginning of the head to the end of the scaly coat) and carcass weight (CW, weight after removing the gills, viscera, and scales). We did not consider the sex of the fish, as they did not have visibly differentiated gonads, and there was little difference in growth rate between males and females. Tail fins from each individual were collected and preserved in anhydrous ethanol at 4 °C for DNA extraction.

### 2.2. Methods

#### 2.2.1. Extraction of Genomic DNA, Library Construction, and Whole Genome Resequencing

DNA was extracted from preserved fin clips using the GenoPrep Polysaccharide Polyphenol DNA Extraction Kit (Molbreeding Biotechnology Co., Ltd., Shijiazhuang, China). The quantity and quality of the DNA were evaluated using the Qubit 2.0 Fluorometer (Thermo Fisher Scientific, Waltham, MA, USA) and 1.5% agarose gel electrophoresis, respectively. Resequencing libraries were constructed from quality-checked DNA using the GenoBaits DNA Library Prep Kit for ILM kit (Molbreeding Biotechnology Co., Ltd., Shijiazhuang, China). Sequencing was performed on the BGI MGI-2000/MGI-T7 platform using the PE150 mode.

#### 2.2.2. Genotyping and Quality Control

FASTP [25] was used to filter the raw sequencing data obtained from the sequencing platform, with quality control including (a) removing adapter sequences and paired reads containing more than 10 N bases (b) deleting paired reads in which the number of low-quality bases (Q ≤ 20) exceeds 40% of the read length. Then, the clean reads were aligned against the reference genome of *L. maculatus* using the BWA-MEM algorithm in the BWA software (v 0.7.15) [26]. The reference genome was downloaded from the Figshare database (https://figshare.com/articles/dataset/Draft_genome_of_the_Chinese_seabass_Lateolabrax_maculatus_/7405694, accessed on 10 January 2023), which is an improved version compared to the one in the GenBank database (GenBank accession: GCA_004028665.1) [27]. Variants were detected by GATK (v 4.0.5.1) [28]. Quality control was performed using PLINK (v 1.9) [29] with the following criteria: --geno 0.2, --hwe 0.00001, --maf 0.05. Missing genotypes were imputed by Beagle (v 4.1) [30] to improve the completeness and accuracy of the genotyping data. Retained SNPs were annotated by SnpEff (v 5.2) [31].

#### 2.2.3. Genome-Wide Association Analysis

The analysis of linkage disequilibrium (LD) decay was performed by PopLDdecay (v3.41) [32]. The population structure of the samples was analyzed using Admixture (v1.3.0) [33]. GWAS was conducted based on three models—MLM [21], Blink [23], and FarmCPU [24] implemented in the R package GAPIT (v 3.0) [34]. Significance thresholds in GWAS were determined by Bonferroni correction, which adjusts the significance threshold based on the number of independent tests, i.e., the number of SNPs used in GWAS, such as 1/N or 0.05/N. Considering that using all SNPs for correction can result in an excessively high significance threshold, reducing the chances of discovering true associations [35], we used the number of independent SNPs obtained after LD filtering with PLINK (v1.9) [29] (parameters: --indep-pairwise 150 10 0.2) as the N value for Bonferroni correction. The significance threshold and the suggestive significance threshold for GWAS were set at 0.05/N and 1/N, respectively [3]. The Kruskal–Wallis Test or Wilcoxon Test was used to analyze the correlations between SNP genotypes and growth traits. Specifically, the Kruskal–Wallis Test was applied to SNPs with three genotypes, while pairwise significance levels were tested using the Wilcoxon Test for SNPs that had only two genotypes [36,37]. The results were further visualized using R package ggplot2. The SNP distribution heat maps, Manhattan plots, and quantile–quantile (Q–Q) plots were drawn by the R package CMplot [38].

GCTA (v 1.94.1) [39] was used to calculate the *PVE* for each SNP. *PVE* was calculated using the formula shown below.

*PVE* = [2 ∗ (*beta*^2^) ∗*MAF* ∗ (1 − *MAF*)]/[2 ∗ (*beta*^2^) ∗ *MAF* (1 − *MAF*) + ((*se*(*beta*))^2^) ∗ 2 ∗ *N* ∗ *MAF* ∗ (1 − *MAF*)]

*N* represents the total number of samples containing the SNP information, *se*(*beta*) is the standard error of the effect size for the genetic variant, *beta* is the effect size for the genetic variant, and *MAF* is the minor allele frequency for the genetic variant.

#### 2.2.4. Identification and Analysis of the Candidate Genes Related to Growth

Genes were considered growth-related candidates when significant loci were located within the genes or were the closest loci within 1 Mb. Variants were annotated by SnpEff (v 5.2) [31]. The sequences of candidate genes were extracted by bedtools [40] and annotated by blastp against the nr database in the National Center for Biotechnology Information (https://www.ncbi.nlm.nih.gov/, accessed on 12 August 2023). The R package ClusterProfiler (v4.12.6) [41] was employed to conduct functional enrichment analyses of the Kyoto Encyclopedia of Genes and Genomes (KEGG) pathways. Pathway assignment was achieved by submitting the protein sequences of the candidate genes to the KEGG Automatic Annotation Server (KAAS, https://www.genome.jp/tools/kaas/, accessed on 2 October 2024). The *p*-values were corrected to control the false discovery rate (FDR) using the Benjamini and Hochberg (BH) method [42]. Pathways with a *q*-value < 0.05 were defined as significant. Additionally, protein–protein interaction (PPI) network and PPI enrichment analysis were carried out using Metascape [43]. Briefly, PPI enrichment analysis was performed using STRING [44], BioGrid [45], OmniPath [46], and InWeb_IM [47]. Only physical interactions in STRING (physical score > 0.132) and BioGrid were used [43]. Gene Ontology (GO) enrichment analysis was applied to extract “biological meanings” from the network component, where the top three best *p*-value terms were retained by default [43].

#### 2.2.5. Tissue Expression Profile Analysis of the Growth-Related Candidate Genes by Quantitative Real-Time Polymerase Chain Reaction (qRT-PCR)

A total of 12 kinds of tissues, including brain, head, kidney, gut, gill, eyes, liver, spleen, heart, kidney, blood, skin, and muscle, were collected from three healthy fish. The tissue expression profiles of *PTPRA*, *SLC7A8*, *PARK2*, *SORCS2*, and *ZNF436* were determined by qRT-PCR. These five candidate genes were selected because the *PVE* of their corresponding SNPs reached 10%, and they were also related to metabolism or development. The total RNA extraction, synthesis of first-strand cDNA, and subsequent qRT-PCR followed our previous report [48]. The relative expression levels of target genes were normalized to β-actin using the 2^−ΔΔCt^ method and expressed relative to their levels in muscle [49,50].

## 3. Results

### 3.1. Selection of Fast-Growing and Slow-Growing Individuals

**Figure 1:**
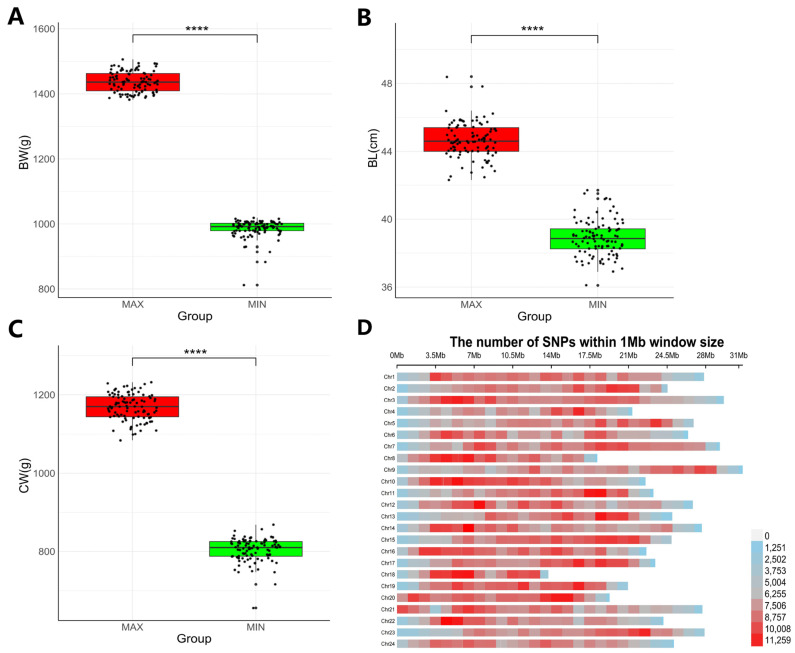
The comparison of growth traits between the fast-growing and slow-growing *L. maculatus* and the distribution of the SNPs used for GWAS on the chromosomes. (**A**–**C**) The comparison of body weight (**A**), body length (**B**) and carcass weight (**C**) between the 100 fast-growing *L. maculatus* and the 100 slow-growing ones. (**D**) The distribution of SNPs used for GWAS on the chromosomes. The redder the color, the more SNPs within a 1 Mb region; the bluer the color, the fewer SNPs. **** refers to *p* value < 0.0001.

Two groups of *L. maculatus* with significant differences were selected from the same breeding population, comprising 100 slow-growing individuals with body weights ranging from 812 to 1019 g and 100 fast-growing ones with body weights from 1382 to 1506 g. The BW, BL, and CW of each individual are shown in Table S1. The slow-growing individuals had a body weight of 985.63 ± 27.94 g, body length of 38.874 ± 1.059 cm, and carcass weight of 803.6 ± 30.395 g, while the fast-growing individuals had a body weight of 1437.200 ± 31.303 g, body length of 44.644 ± 1.038 cm, and carcass weight of 1167.870 ± 33.504 g. There were highly significant differences between the two groups in weight, body length, and carcass weight (Figure 1A–C).

### 3.2. Genotyping

Whole-genome resequencing was performed on 200 individuals of *L. maculatus*. Of the 200 individuals, 184 were sequenced with an average Q30 of 95.21% (Table S2), an average depth of 4.526×, and an average alignment rate of 98.91% (Table S3). The other sixteen individuals underwent high-depth sequencing, with an average Q30 of 97.62% (Table S2), an average depth of 14.623×, and an average alignment rate of 98.79% (Table S3). A total of 19,504,127 SNPs, averaging 97,520 SNPs per individual, were detected by GATK, and 4,561,929 SNPs were retained after filtering. Following the imputation of missing genotypes, we obtained a total of 4,528,936 SNPs, which were evenly distributed across all chromosomes without large gaps (Figure 1D), and the average distance between SNPs was 175.8 bp. These SNPs were used in subsequent GWAS analyses.

### 3.3. Genome-Wide Association Analysis

**Figure 2:**
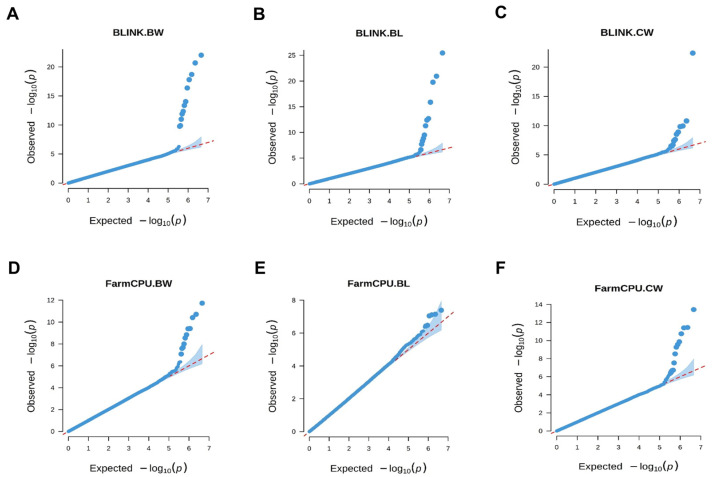
Q–Q plot for body weight, body length, and carcass weight based on the Blink and FarmCPU model. (**A**) Quantile–quantile plot for body weight based on the Blink model. (**B**) Quantile–quantile plot for body length based on the Blink model. (**C**) Quantile–quantile plot for carcass weight based on the Blink model. (**D**) Quantile–quantile plot for body weight based on the FarmCPU model. (**E**) Quantile–quantile plot for body length based on the FarmCPU model. (**F**) Quantile–quantile plot for carcass weight based on the FarmCPU model.

**Figure 3:**
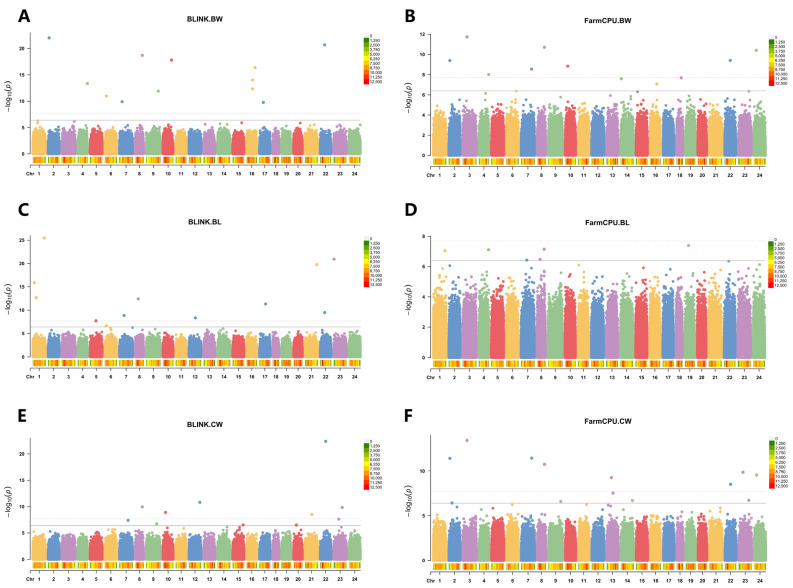
Manhattan plot for the GWAS of body weight, body length, and carcass weight in *L. maculatus*. (**A**,**B**) Manhattan plot of GWAS for body weight based on the Blink (**A**) and FarmCPU (**B**) models. (**C**,**D**) Manhattan plot of GWAS for body length based on the Blink (**C**) and FarmCPU (**D**) models. (**E**,**F**) Manhattan plot of GWAS for carcass weight based on the Blink (**E**) and FarmCPU (**F**) models.

The LD decay analysis showed that the LD decay distance of the *L. maculatus* genome in this population is 120 bp when the squared correlation coefficient (r^2^) decreased to 0.1 (Figure S1). The genetic structure analysis indicated that the cross-validation error rate was lowest when k = 1 (Figure S2), suggesting that there is no obvious population structure among the *L. maculatus* samples and that the samples are suitable for subsequent GWAS. Additionally, the models of MLM, Blink, and FarmCPU were evaluated using Q–Q plots. The Q–Q plots for body weight, body length, and carcass weight based on Blink and FarmCPU have clear upward tails, which means the points in the lower left corner are on the diagonals, while the points in the upper right corner are above the diagonals (Figure 2). This suggested that FarmCPU and Blink can effectively reduce false positives and false negatives. In contrast, the Q–Q plots based on the MLM model showed that all observed *p*-values were not significantly greater than the expected ones, indicating that no significant loci were found (Figure S3). GWAS based on Blink and FarmCPU identified 50 significant markers associated with growth, composed of 19 related to body weight, 17 to body length, and 23 to carcass weight (Figure 3). Two markers were shared by the three growth-related traits (Figure S4).

**Figure 4:**
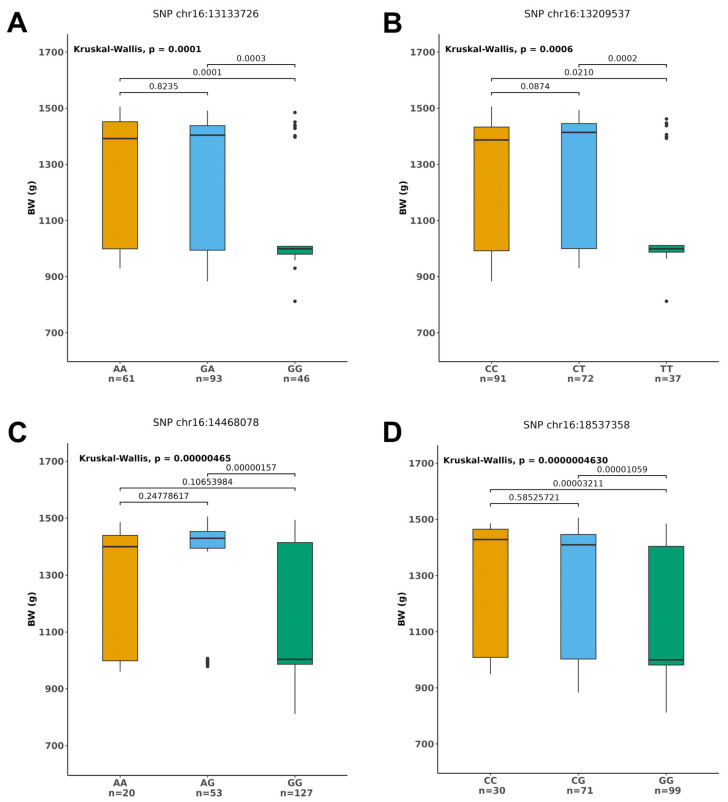
Boxplot of body weight for different genotypes of *L. maculatus*. The *y*-axis represents the body weight of the individuals with different genotypes, and different colors indicate different genotypes, with the “n” representing the number of individuals for each genotype. (**A**) Body weight of individuals with different genotypes for the SNP on chr16:13133726; (**B**) Body weight of individuals with different genotypes for the SNP on chr16:13209537; (**C**) Body weight of individuals with different genotypes for the SNP on chr16:14468078; (**D**) Body weight of individuals with different genotypes for the SNP on chr16:18537358.

*PVE* was calculated for each of the significant markers. The *PVE* ranges from 0.072%–15.82% (Table 1). Additionally, there are significant differences among different genotypes in body weight, body length, or carcass weight. For the four SNPs located on chromosome 16—chr16:13133726, chr16:13209537, chr16:14468078, chr16:18537358, there are highly significant difference among the individuals with different genotypes in body weight (Figure 4). For example, the body weight of the individuals with genotype AA and GG at the chr16:13133726 locus was significantly higher than those with genotype GA (Figure 4A). Additionally, for the four SNPs located on the chromosomes 5, 7, 8, and 21—chr5:12828690, chr7:10323545, chr8:8491406, chr21:25857252, there are highly significant differences among different genotypes in body length (Figure S5). Similarly, for the four SNPs—chr7:19065016, chr10:5554722, chr22:9945779, chr23:11019710—there were also highly significant differences among different genotypes in carcass weight (Figure S6).

Of the 50 markers, 26 (52%) were located in intron, 12 (24%) in the intergenic region, 11 (22%) in upstream and downstream regions of the genes, and one (2%) in the exon region. The variation in the exon was caused by a SNP, which contributed to about 12% *PVE* and led to a nonsynonymous mutation in the *ZNF436*. *ZNF436* is a transcription factor and involved in lipid metabolic processes.

### 3.4. Candidate Gene Identification and Analysis

**Figure 5:**
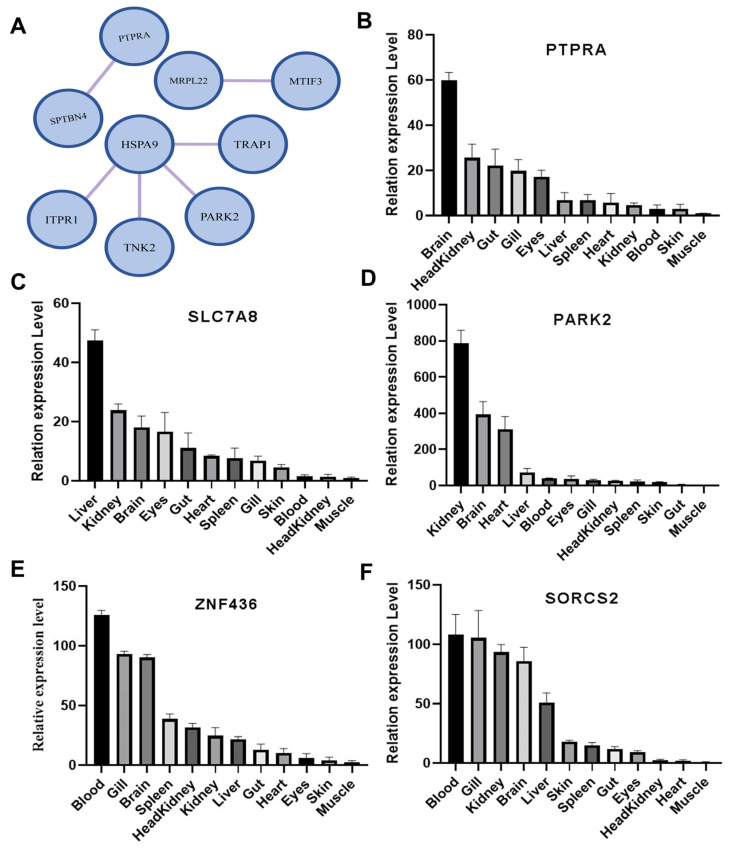
Protein–protein interaction network and tissue expression profile analysis of the growth-related candidate genes. (**A**) The protein–protein interaction network of the growth-related candidate genes. (**B**–**F**) Tissue expression profile analysis of *PTPRA* (**B**), *SLC7A8* (**C**), *PARK2* (**D**), *ZNF436* (**E**), and *SORCS2* (**F**).

Furthermore, a total of 47 candidate genes were annotated. These genes function primarily metabolism of energy, glucose, and lipid, and the development of musculoskeletal and nervous systems. Some of these genes, such as *BCAS3*, *ITPR1b*, *SLC7A8*, *MTIF3*, *SLC38A10*, and *PARK2* have been proved to be associated with growth by gene knockout or knockdown in mice or zebrafish (Table 1). The KEGG enrichment analysis identified only one significant pathway: the phosphatidylinositol signaling system. Additionally, a PPI network was constructed by Metascape, and the network is mainly enriched in the neuromuscular process, mitochondrial transport, and Parkinson’s disease (Figure 5A).

**Table 1:** Summary of the candidate markers and genes associated with growth-related traits.

Locus	Allele	*MAF*	Location	Trait and *PVE*%	Candidate Gene	Gene Function
chr1:3532952	G/A	0.09	Intron	BL-9.46	*SPTBN4*	Frameshift mutation causes congenital myopathy, characterized by a lack of type I muscle fibers [51,52]
chr1:7536990	C/A	0.1075	Intron	BL-3.47	*ROBO2*	Mediating the connection between muscles and corresponding tendons, and remodeling embryonic muscles [53]
chr1:25193935	A/ACAATTTT	0.4825	Intron	BL-0.65	*BCAS3*	Cardiovascular remodeling, its loss causes a global developmental delay [54,55]
chr2:2181025	TC/T	0.1325	Intron	BW-9.18; CW-8.12	*C5orf22*	Component of the WBP11/PQBP1 splicing complex [56]
chr2:6750577	C/T	0.0525	Intron	CW-0.99	*tnk2b*	Unknown
chr3:9167600	T/C	0.3225	Intergenic	BW-6.20; CW-6.48	*NR3C2*	Inhibiting angiogenesis [57,58]
chr4:21198683	T/C	0.075	Intron	BL-5.92; BW-8.18;	*PIEZO2*	Essential for mammalian proprioceptive mechanotransduction [59]
chr5:12828690	G/T	0.265	Intron	BL-1.99	*KIFC3*	A microtubule minus end-directed motor [60]
chr6:3003165	A/G	0.37	Intron	BL-0.96	*XKR7b*	Unknown
chr6:3479789	C/T	0.12	Intron	BW-1.54	*GDAP1L1*	Involved in mitochondrial synthesis and dynamic regulation [61]
chr7:5863464	GT/G	0.1125	Intron	BW-1.80	*ITPR1b*	Cranial bone development, *ITPR1b* knockdown could remarkably reduce craniofacial bone formation in zebrafish [62]
chr7:10323545	T/G	0.31	Intron	BL-3.26	*RAP1GAPB*	Inhibiting GDNF/Ret-induced neurite outgrowth [63]
chr7:12496062	GT/G	0.2325	Intergenic	BL-5.99	*CDH22*	Central nervous system development [64]
chr7:19065016	A/G	0.24	upstream	CW-6.42	*IP6K2b*	Unknown
chr7:22666212	T/C	0.075	downstream	BW-6.22; CW-6.61	*PLXNA1*	Acting as a guidance receptor for developing axons toward their targets [65]
chr8:6470380	T/C	0.0675	Intergenic	BL-10.24	*SLC7A8*	Implicated in fat accumulation and glucose metabolism, *SLC7A8* knockout significantly reduces mouse body weight [66]
chr8:6491406	A/C	0.1225	Intron	BL-5.53	*SLC7A8*	The same gene as the previous line
chr8:15408385	C/T	0.145	Intergenic	BL-15.82; BW-13.61; CW-13.00	*SORCS2*	Required for motor neuron diversification and axon outgrowth [67]
chr9:23160296	G/A	0.11	Intron	CW-5.98	*PDE8A*	Regulating production of steroid hormones such as testosterone and progesterone [68,69,70]
chr9:26900462	G/A	0.07	Intron	BW-4.01	*LRP4*	Crucial for the formation and maintenance of muscle spindles and the formation of neuromuscular junctions [71,72]
chr9:26976752	A/G	0.0675	Intron	CW-8.77	*LRP4*	The same gene as the previous line
chr10:5258207	AATT/A	0.11	Intergenic	BW-2.82	Hypothetical proteins	Unknown
chr10:5554722	G/A	0.295	Intron	CW-7.07	*MTIF3*	Related to transcription of mitochondrial mRNA and coordinated assembly of oxidative phosphorylation complexes, *MTIF3* knockout could decreases body weight and reduces skeletal muscle fiber size [73]
chr10:19011581	C/T	0.0625	Intergenic	BW-0.89	*CNIH3*	Involved in hippocampal learning, CNIH3-knockout mice show severe memory deficits [74]
chr12:14017562	T/C	0.045	Intergenic	BL-3.84	*pcdh2ac*	Ionic transport and physiology, also a candidate gene related to growth in common carp [75]
chr12:24036447	CTGTTTCT	0.1875	Intergenic	CW-1.34	*HSPA9*	Related to apoptosis and the permeability of the mitochondrial membrane [76]
	TTTCTAT/C					
chr13:9885815	TA/T	0.1575	Intergenic	CW-1.92	*IKZF2*	Lymphocyte development and apoptosis, and highly expressed in hematopoietic stem cells [77,78]
chr13:13529327	C/CA	0.44	upstream	CW-1.79	-	-
chr14:1346742	C/CA	0.43	Intron	BW-2.40	*DGKZb*	Unknown
chr14:24885400	A/G	0.085	Intron	CW-3.88	*Spire2*	Involved in actin filaments and host immunity [79]
chr15:22822005	T/TA	0.475	Intergenic	CW-0.07	*MRPL22*	A mitochondrial ribosomal protein
chr16:13133726	G/A	0.4625	Intron	BW-4.76	*BCR*	B cell antigen receptor [80]
chr16:13209537	C/T	0.365	Intron	BW-1.56	*rimbp2a*	Unknown
chr16:14468078	A/G	0.2325	Intergenic	BW-6.08	*HARBI1*	A domesticated transposase-derived protein [81]
chr16:18537358	C/G	0.3275	nonsynonymous	BW-12.81	*ZNF436*	Intrinsic regulators of lipolysis, *ZNF436* knockdown inhibited adipocyte lipolysis [82]
chr17:8541064	T/A	0.0525	downstream	BW-2.52	*STK17A*	Related to apoptosis [83]
chr17:13371860	A/C	0.2	downstream	BL-0.56	*TRAP1*	Regulating cellular energy metabolism through interactions with components of the tricarboxylic acid cycle and oxidative phosphorylation system [84,85,86]
chr18:10051109	T/G	0.08	downstream	BW-6.21	*RBP43*	Unknown
chr19:7485902	C/T	0.0525	upstream	BL-11.30	*SLC38A10*	SLC38A10-knockout mice display a decreased body weight and an increased risk-taking behavior [87]
chr20:5933315	T/A	0.0725	Intron	CW-3.14	*SOX5*	Mediated chondrogenesis and chondrocyte differentiation, patient with idiopathic short stature carried with heterozygous novel variant in *SOX5* [88], The candidate gene related to growth in *Micropterus salmoides* [89]
chr21:14975526	C/A	0.115	upstream	CW-1.71	*PLEKHA2*	The lipid metabolism mediator [90]
chr21:25857252	A/G	0.2275	upstream	BL-8.17	*LMO4b*	Participating in adipocyte proliferation [91]
chr22:9945779	T/C	0.3225	Intron	BL-11.64; BW-12.37; CW-12.44	*PARK2*	*PARK2* is a lipid-responsive regulator of fat uptake. *PARK2*-knockout mice display reduced body weight under an equicaloric high-fat and -cholesterol diet [92].
chr22:12090488	G/C	0.0525	Intron	BW-13.73; CW-14.11	*RPAP1*	Related to cell differentiation and viability [93]
chr23:520106	C/T	0.145	Intron	BL-10.23	*PTPRA*	Female mice genetically lacking *PTPRA* exhibit reduced weight and adiposity and increased energy expenditure [94,95], also involved in neurodevelopment [96]
chr23:9111788	G/GGT	0.485	downstream	CW-0.62	*TMEM161A*	Regulating bone formation and bone strength [97]
chr23:11019710	G/A	0.21	Intron	CW-6.20	*GLIS1*	Induces multi-level epigenetic and metabolic remodelling in stem cells that facilitates the induction of pluripotency [98]
chr23:18515222	G/C	0.0625	Intron	CW-8.49	*KANK3*	Involved in vascular vessel development [99]
chr23:21934479	C/T	0.05	downstream	CW-0.98	*GPSM2*	Maintains cell polarization and regulates the cell cycle [100]
chr24:5189973	CACAG/C	0.095	Intergenic	BW-5.79; CW-6.14	*MEIS1b*	Inhibit erythroid progenitor development and sustain general hematopoietic cell proliferation [101,102]

#### Tissue Expression Profile Analysis of the Growth-Related Candidate Genes

The tissue expressions of the *PTPRA*, *SLC7A8*, *PARK2*, *SORCS2*, and *ZNF436* were assessed by qRT-PCR. All five genes are constitutively expressed in all tissues examined, with the lowest levels found in muscle. The highest mRNA profile of the *PTPRA*, *SLC7A8*, *PARK2* and *ZNF436* were found in the brain, liver, kidney and blood, respectively (Figure 5B–E). The mRNA expression of *SORCS2* is mainly found in blood, gills, kidneys, and the brain (Figure 5F).

## 4. Discussion

### 4.1. XP-GWAS Combined with High-Density Genotypes from Whole-Genome Sequencing Identifies Growth-Related Markers

Sample size and the density of genomic markers are two important factors that significantly impact the accurate localization of the target traits in GWAS. Sample size is very important for ensuring enough statistical power in GWAS. The larger the sample size, the more reliable the GWAS results and the more associated loci, while larger sample sizes come with higher costs [103,104]. The complexity of the target trait and sample heterogeneity are two main factors affecting the determination of optimal sample size. For conventional GWAS analysis, no less than 200 is enough for the traits that are controlled by 1–2 major genes and have rich phenotypic variation [104,105,106], while the sample size should be increased for GWAS of polygenic traits with small phenotypic differences, especially when the traits are probably controlled by genes with low-frequency alleles [104,105]. Five hundred individuals are sufficient to detect a QTL (Quantitative Trait Locus) explaining 5% of the phenotypic variance if the polygenic contribution is 50% of the phenotypic variance. Therefore, conventional GWAS analysis requires large-scale genotyping and considerable sequencing costs, making GWAS an expensive analytical approach. To reduce costs, XP-GWAS was developed, in which only the samples with extreme phenotypes are sequenced. Empirical studies have shown that XP-GWAS outweighs conventional GWAS by reducing the number of individuals required to be genotyped [22,107]. At the same time, the resolution afforded by XP-GWAS is comparable with the results from conventional GWAS [22,107]. Although there is still a lack of studies on the sample size required for genotyping in XP-GWAS, which is expected to be affected by many factors, including selection intensity, sample size, and the precision of phenotyping, the sample sizes genotyped in XP-GWAS in previous reports mainly range from 36 to 140 [107,108,109,110,111,112]. For example, 40 individuals, including 20 thermal-tolerant and 20 thermal-sensitive individuals of *L. crocea*, were sequenced for XP-GWAS of the thermal tolerance trait [108]. A total of 120 individuals, with 60 ammonia-sensitive and 60 ammonia-tolerant individuals, were genotyped for XP-GWAS of ammonia nitrogen tolerance trait [109]. Eighty-six and 85 individuals were sequenced for XP-GWAS of the apple red flesh color coverage and internal flesh browning disorder traits, respectively [107]. Seventy-two individuals were selected for sequencing for XP-GWAS to identify the loci affecting the caffeine and trigonelline content of *Coffea arabica* beans, with 36 individuals for each of the traits [110]. In this study, 200 individuals, a sample size that far exceeds most of those reported previously, with significant differences in growth rate, are sequenced. The increased sample size could enhance the power to detect trait-associated variants using XP-GWAS. High-density markers are essential for fine mapping of the target traits. Common genotyping methods include SNP chips, reduced-representation genome sequencing, and whole-genome low-coverage sequencing [113]. The latter often combines genotype imputation to obtain high-density variants at the whole-genome level [113,114]. Therefore, we utilized XP-GWAS combined with low-coverage sequencing technology to identify markers associated with growth traits in *L. maculatus*. The samples for GWAS consisted of two groups of *L. maculatus* with significant differences in growth traits (Figure 1A–C). For genotyping, 19.5 million SNPs were obtained by whole-genome sequencing, and approximately 4.53 million SNPs were used for GWAS analysis after filtering. These SNPs were evenly distributed across all chromosomes without large gaps (Figure 1D), and the average distance between SNPs was only 175.8 bp. The high-density markers and XP-GWAS underlined the fine mapping of growth traits.

### 4.2. XP-GWAS Based on Three Models Identified 50 Growth-Related Markers with the Highest PVE up to 15.82%

Two types of Manhattan plots in GWAS have been proposed [24,115]. One is the New York City (NYC) type Manhattan plot, which is derived from the full skyline view of NYC and is used to describe how the concentrated distribution of the markers’ *p*-values looks like tall buildings [115]. The other is the Kansas-type Manhattan plot. The Manhattan here does not refer to the one in NYC, but rather to a small town in Kansas, where there are few tall buildings. The highest human-made objects may be the helicopters. The Kansas-type Manhattan plots are used to depict the scattered associated markers that appear like helicopters flying over Manhattan in Kansas [115]. In this study, significant markers appear as isolated peaks, which means that the Manhattan plot belongs to the Kansas type. The reasons may mainly be caused by the rapid decay of LD in the *L. maculatus* genome and the choice of multiple-loci models. The concentrated distribution of the significant markers’ *p*-values in the NYC-type Manhattan plot results from the linkage disequilibrium among the significant loci. However, the LD decay distance of the *L. maculatus* genome in this population is only about 120 bp (Figure S1) when the r^2^ declines to 0.1, which is very close to a previous report (about 150 bp) [3]. This suggests that the genetic linkage of adjacent SNPs is very weak, and the trait-related loci will tend to appear as isolated peaks. The single-locus model of MLM and two multiple-loci models (FarmCPU and BLINK) are commonly used models in GWAS. Nevertheless, several studies have suggested that the multiple-loci models, FarmCPU and BLINK, are superior to MLM in identifying trait-associated markers [116,117,118]. FarmCPU and BLINK can effectively deal with false positives and false negatives, while MLM commonly produces false negatives. In this study, we assessed these three models by Q–Q plots; many significant loci were identified by FarmCPU and BLINK but not by MLM (Figure 2 and Figure S3), which supports the previous conclusion. Thus, only the multiple-loci models, FarmCPU and BLINK, were used for the identification of growth-related loci in this study. In the multiple-loci model, the Manhattan plots manifest Kansas-type rather than the NYC-type, as only one marker in an LD block can have a significant *p*-value [24,115]. Consistently, Manhattan plots created by FarmCPU and BLINK in the previous reports are Kansas-type, and the significant loci are isolated rather than concentrated like skyscrapers [23,24,36,116,119,120,121,122].

Marker-assisted breeding can improve breeding accuracy and shorten the time needed for developing new fish varieties. However, it is essential to consider the species and populations to which the markers are applicable, as well as the extent to which these markers influence phenotypic variation in the selective breeding program. On one hand, markers developed based on a particular genetic background may not be applicable to another species or population, because allele frequencies and phenotypes may vary greatly among species and populations [1,123]. Therefore, although many growth-related markers have been developed in the last few decades in various fish species, the applicability of these markers in the *L. maculatus* breeding program is still awaiting clarification. It is worth mentioning that recently Zhang et al. [3] performed GWAS on 512 individuals of *L. maculatus* from two different populations (DongYing population and Tangshan population) with growth traits following a near-normal distribution, and identified 66 growth-related SNPs based on the 3.75 million SNPs and MLM model. However, there was no overlap between the SNPs identified in our study and those identified by Zhang et al. Although the exact reasons remain unclear, the model selection, population-genetic factors, and SNP density can influence the GWAS results [123,124,125]. The highest *PVE* value of the markers identified in this study is 15.82%, a value much higher than the *PVE* values reported previously in other fish, such as catfish (<6.72%) [4], Atlantic salmon (<12%) [19], and rainbow trout (<0.18%) [17]. Additionally, these SNPs were significantly associated with body weight, body length, or carcass weight (Figure 4, Figures S5 and S6). The identification of the growth-related markers will help the breeding of fast-growing varieties of *L. maculatus*.

### 4.3. The Metabolism of Energy, Glucose, and Lipid May Fuel the Growth of L. maculatus

Growth is a polygenic trait and influenced by multiple physiological pathways that regulate metabolism and development [126,127]. In this study, a total of 47 genes were identified as growth-related candidate genes. These genes are mainly involved in the metabolism of energy, glucose, and lipid, and the development of musculoskeletal and nervous systems. Metabolism and growth are two closely related physiological processes. Metabolism can profoundly affect growth by providing nutrients and energy [126,127]. At least 9 genes are related to glucose, lipid, and energy metabolism, including *MTIF3*, *TRAP1*, *GDAP1L1*, *PTPRA*, *SLC7A8*, *PLEKHA2*, *LMO4b*, *PARK2*, and *ZNF436*. *MTIF3* is required for transcription and regulation of translation initiation of mitochondrial mRNA, and the coordinated assembly of oxidative phosphorylation complexes. *MTIF3* knockout significantly decreases body weight and reduces skeletal muscle fiber size in mice [73]. *TRAP1* regulates cellular energy metabolism by interacting with components of the tricarboxylic acid cycle and the oxidative phosphorylation system [84,85,86]. *GDAP1L1* is related to mitochondrial synthesis and dynamic regulation [61]. *PTPRA* is related to the regulation of body weight; female mice genetically lacking *PTPRA* exhibit reduced weight and adiposity, and increased energy expenditure [95]. Additionally, *PTPRA* is also involved in various neurodevelopmental processes [96]. Consistently, the highest level of PTPRA mRNA was present in the brain of *L. maculatus* (Figure 5B). *SLC7A8* is implicated in fat accumulation and glucose metabolism; *SLC7A8* knockout significantly reduces body weight of mice [66]. In *L. maculatus*, the mRNA level in the liver is much higher than in other tissue (Figure 5C). This may be because the liver is of prime importance in carbohydrate and lipid metabolism [128]. *PLEKHA2* is the lipid metabolism mediator [90]. *LMO4b* participates in adipocyte proliferation [91]. *PARK2* is a lipid-responsive regulator of fat uptake [92]. *PARK2* knockout reduces body weight of mice under an equicaloric high-fat and cholesterol diet [92]. *ZNF436* is a member of the zinc finger transcription factor family involved in lipid metabolic processes; *ZNF436* knockdown inhibits adipocyte lipolysis in human adipose-derived stem cells [82]. Here, we detected a SNP in the *ZNF436*, which caused a nonsynonymous mutation and contributed 12% *PVE*. However, the effect of the SNP on growth and the function of *ZNF436* in fishes still await clarification.

### 4.4. The Musculoskeletal Development Contributes to the Growth Performance in L. maculatus

Musculoskeletal system constitutes the main part of a fish and plays a critical role in growth [126]. Here, we identified six candidate genes associated with the development of musculoskeletal systems including *SPTBN4*, *ROBO2*, *LRP4*, *TMEM161A*, *ITPR1b*, and *SOX5*. A frameshift mutation in *SPTBN4* can cause congenital myopathy in humans, characterized by a lack of type I muscle fibers [51,52]. *ROBO2* mediates the connection between muscles and corresponding tendons and plays an essential role in remodeling embryonic muscles [53]. *LRP4* is crucial for the formation and maintenance of muscle spindles and the formation of neuromuscular junctions [71,72]. *TMEM161A* is associated with bone formation and bone strength, the knockout of this gene can promote osteoblast differentiation in mice [97]. *ITPR1b* is involved in the cranial bone development of zebrafish; *ITPR1b* knockdown could remarkably reduce craniofacial bone formation [62]. *SOX5* plays an essential role in chondrogenesis and chondrocyte differentiation, and heterozygous mutations in this gene may result in short stature [88]. Interestingly, *SOX5* is also a candidate gene for growth traits in *Micropterus salmoides*. Altogether, the musculoskeletal development may act as a driver for growth of *L. maculatus*.

### 4.5. Growth Is under the Influence of Genes Regulating Nervous Development

Nervous development could exert a profound influence on growth. For one thing, the nervous system can influence growth by releasing neurotransmitters and neuromodulators [126], such as through the hypothalamic–pituitary–growth hormone axis. For another, environmental stress can also affect growth by nervous system. Here, at least 7 genes were associated with neural development, including *PTPRA, RAP1GAPB*, *PIEZO2*, *CDH22*, *PLXNA1*, *SORCS2*, and *CNIH3*. *RAP1GAPB* functions to inhibit GDNF/Ret-induced neurite outgrowth [63]. *PIEZO2* is essential for mammalian proprioceptive mechanotransduction [59]. *CDH22* plays an important role in development of the central nervous system [64]. *PLXNA1* acts as a guidance receptor for developing axons toward their targets [65]. *SORCS2* is required for motor neuron diversification and axon outgrowth [67]. *CNIH3* is involved in hippocampal learning, and *CNIH3*-knockout mice show severe memory deficits [74]. Additionally, GO enrichment analysis showed that the PPI network of the candidate genes is mainly enriched in neuromuscular processes (Figure 5A). Therefore, our results suggest that genes related to nervous development may have a great influence on the growth of *L. maculates*, although the exact functions of these genes in growth of *L. maculatus* still need further confirmation.

### 4.6. Limitation of Current Study

Compared with conventional GWAS, XP-GWAS is much more powerful in identifying trait-related loci and genes by reducing the number of individuals required to be genotyped without decreasing mapping resolution. Simulated power analyses showed that increasing the numbers genotyped while maintaining selection intensity could enhance the power to detect small-effect QTLs [22,129]. Therefore, although the sample size in this study already has exceeded most of the previous reports in XP-GWAS, more markers will be identified accompanying the increment of the sample size. Additionally, the identified genes are potential candidates for further validation. As with all GWAS studies, the identified loci and genes may not be causative but a result of linkage disequilibrium. Therefore, the functional role of these candidate genes in growth needs to be validated by reverse genetics, such as gene editing.

## 5. Conclusions

Fish are one of the most important sources of high-quality protein. The breeding of fast-growing fish and elucidation of the genetic mechanisms underlying the growth traits have always been popular subjects. The cultivation of fast-growing varieties can increase aquaculture yield and economic benefits, and elucidation of genetic mechanisms lays the foundation for molecular design-based breeding. Here, XP-GWAS combined with high-density genotypes from whole-genome sequencing was used to identify growth-related marker in the *L. maculatus* genome. The average distance between SNPs was only 175.8 bp. The high-density markers and XP-GWAS based on three models identified 50 growth-related markers with the highest *PVE* up to 15.82%, a value much higher than those reported previously in other aquaculture fish. Furthermore, there are significant differences in body weight, body length, or carcass weight among individuals with different genotypes. For example, there is a highly significant difference in body weight among individuals with different genotypes for the four SNPs located on chromosome 16: chr16:13133726, chr16:13209537, chr16:14468078, and chr16:18537358. These markers will help in molecular -assisted breeding of *L. maculatus*. Additionally, a total of 47 genes were annotated as candidates, of which two namely *SORCS2* and *PARK2* were associated with body weight, body length, and carcass weight. The functions of some of these genes in growth have been confirmed in mice or zebrafish through gene knockout or knockdown. The 47 candidate genes function mainly to regulate the metabolism of energy, glucose and, lipid, and the development of musculoskeletal and nervous systems. We discussed the role of metabolism and development in growth of *L. maculatus*. Altogether, our study identified growth-related markers and candidate genes, which will help to develop the fast-growing varieties of *L. maculatus* and elucidate genetic mechanisms underlying the growth traits.

## Data Availability

The raw sequencing reads were deposited in the NCBI SRA database with the BioProject accession PRJNA1138314.

## Supplementary Materials

The following supporting information can be downloaded at https://www.mdpi.com/article/10.3390/ani14202995/s1, Figure S1: LD decay plot of SNPs. Figure S2: The CV value of each K value; Figure S3: Q–Q plot for body weight, body length, and carcass weight based on the MLM model. A: Quantile–quantile plot for body weight, B: Quantile–quantile plot for body length, C: Quantile–quantile plot for carcass weight; Figure S4: Venn of SNPs related to body weight, body length, and carcass weight; Figure S5: Boxplot of body length for different genotypes of *L. maculatus*. The *y*-axis represents the body weight of the individuals with different genotypes, and different colors indicate different genotypes, with the ‘n’ representing the number of individuals for each genotype; Figure S6: Boxplot of carcass weight for different genotypes of *L. maculatus*. The *y*-axis represents the body weight of the individuals with different genotypes, and different colors indicate different genotypes, with the ‘n’ representing the number of individuals for each genotype; Table S1: Body length, body weight and carcass weight of the samples for GWAS; Table S2: Quality of the whole-genome resequencing data of the *Lateolabrax maculatus* samples used for GWAS analysis; Table S3: Statistics of the alignment results after mapping the clean reads to the reference genome of *Lateolabrax maculatus*.
