# Supplementary figures and images for: Extreme-Phenotype Genome-Wide Association Analysis for Growth Traits in Spotted Sea Bass (Lateolabrax maculatus) Using Whole-Genome Resequencing
Source: Animals (Basel). 2024 Oct 17;14(20):2995. doi: 10.3390/ani14202995 (PMC11503831; doi:10.3390/ani14202995)

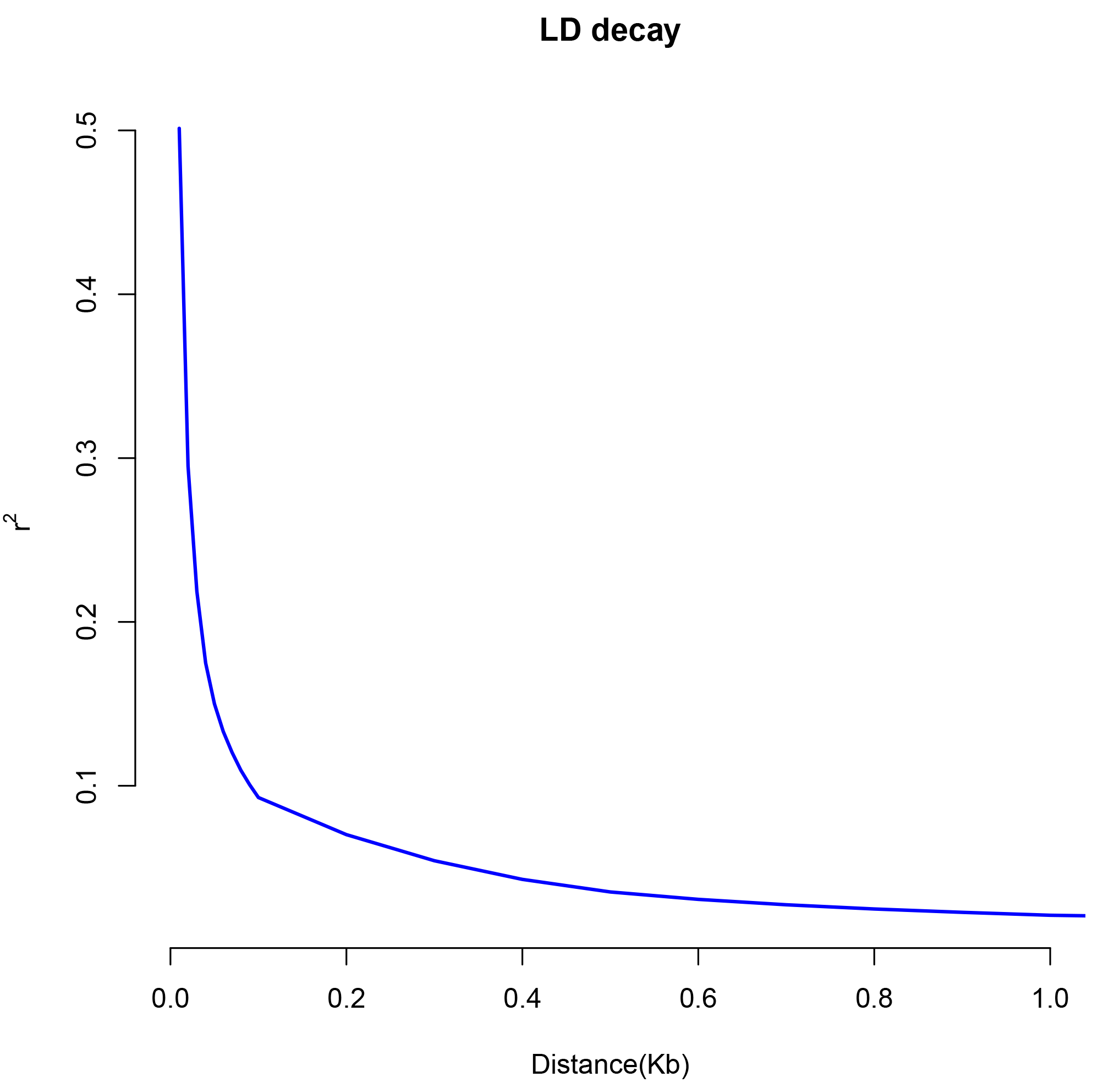

Supplement: Supplementary file 1 [file animals-14-02995-s001.zip › Figure S1.tif]

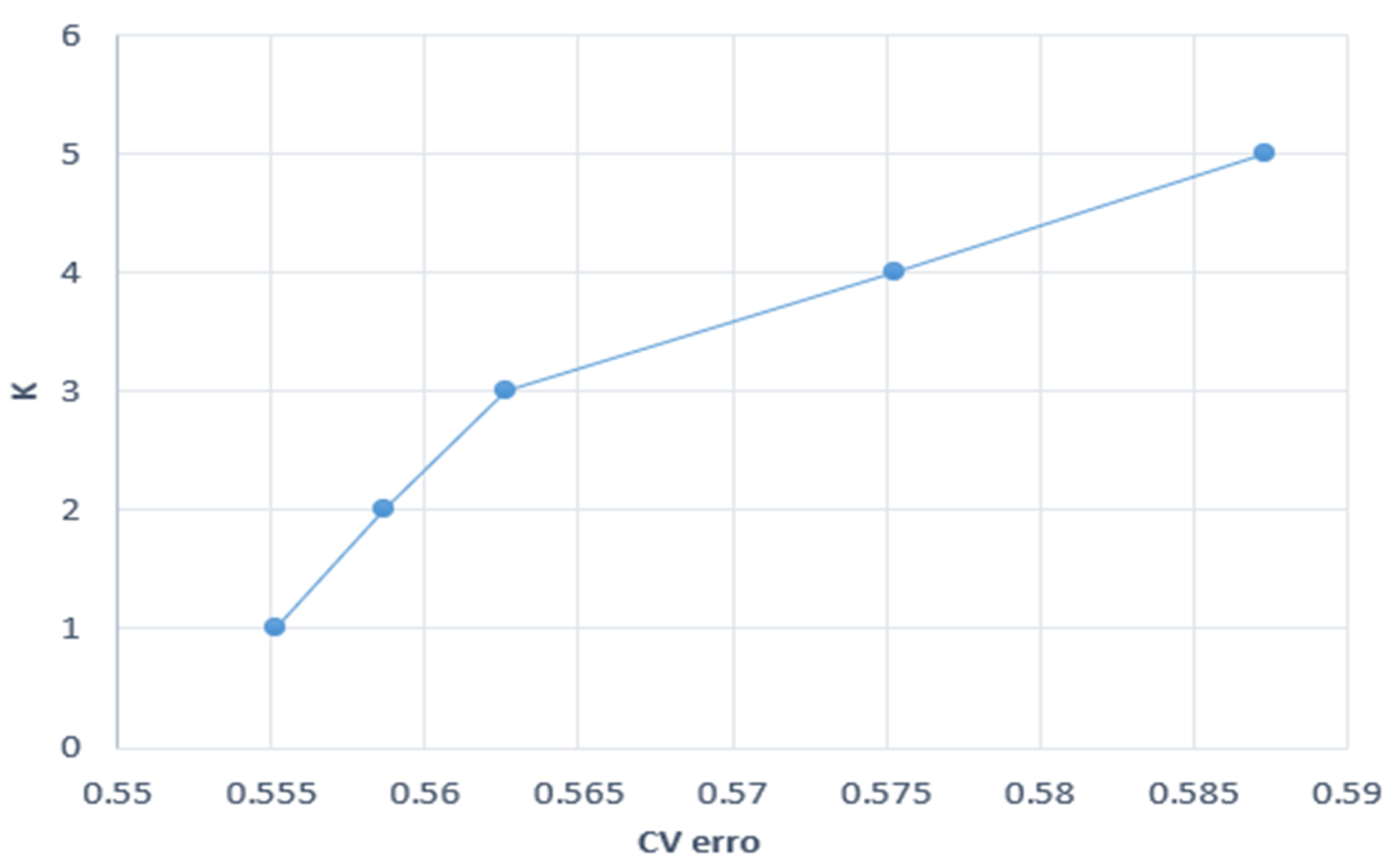

Supplement: Supplementary file 1 [file animals-14-02995-s001.zip › Figure S2.tif]

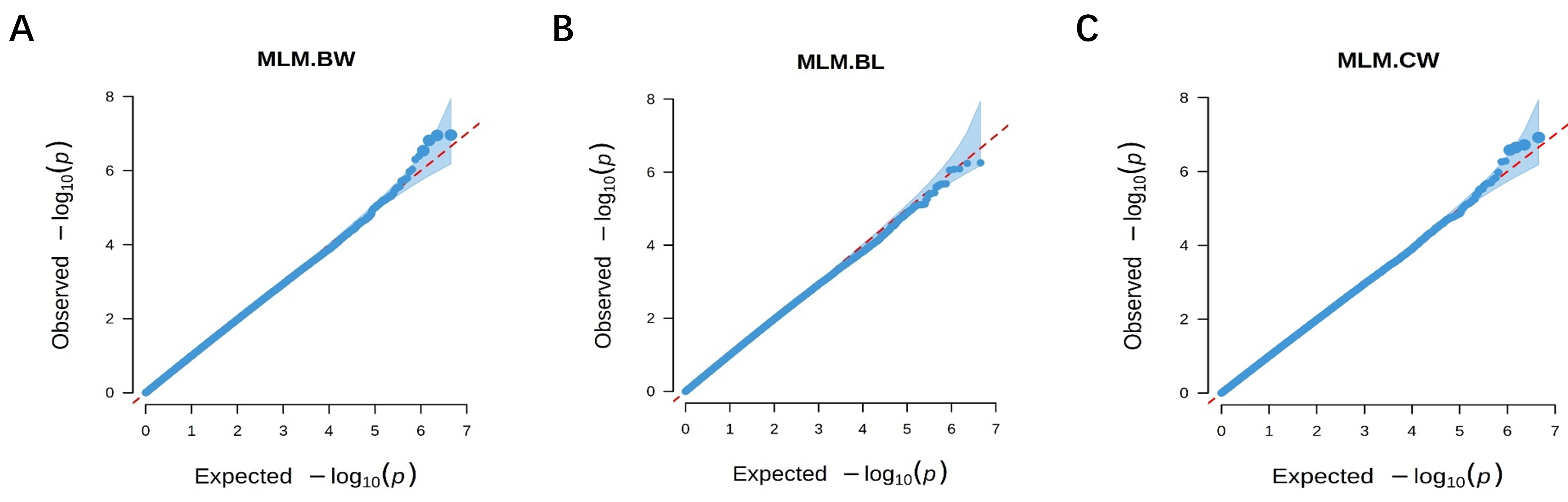

Supplement: Supplementary file 1 [file animals-14-02995-s001.zip › Figure S3.tif]

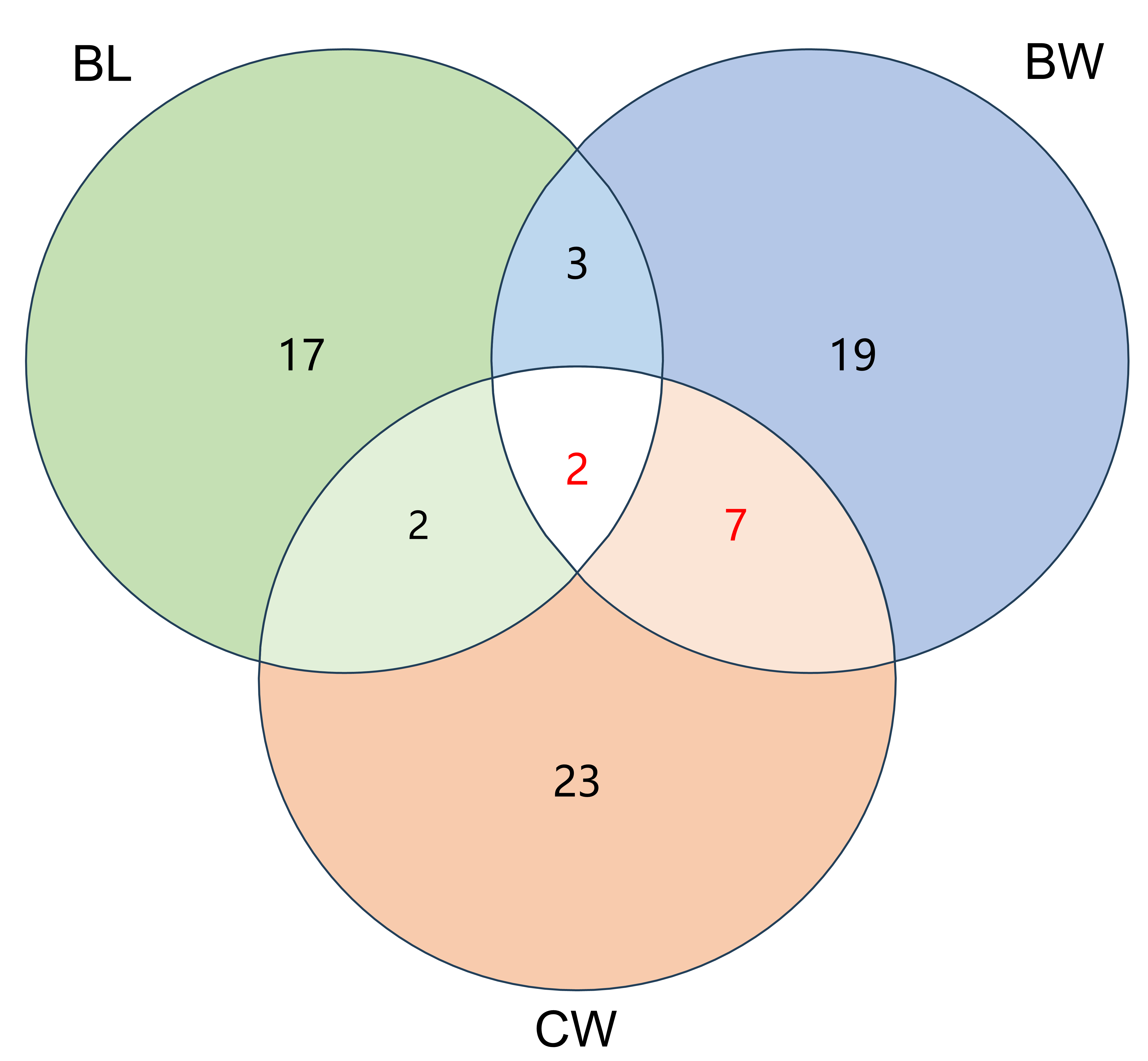

Supplement: Supplementary file 1 [file animals-14-02995-s001.zip › Figure S4.tif]

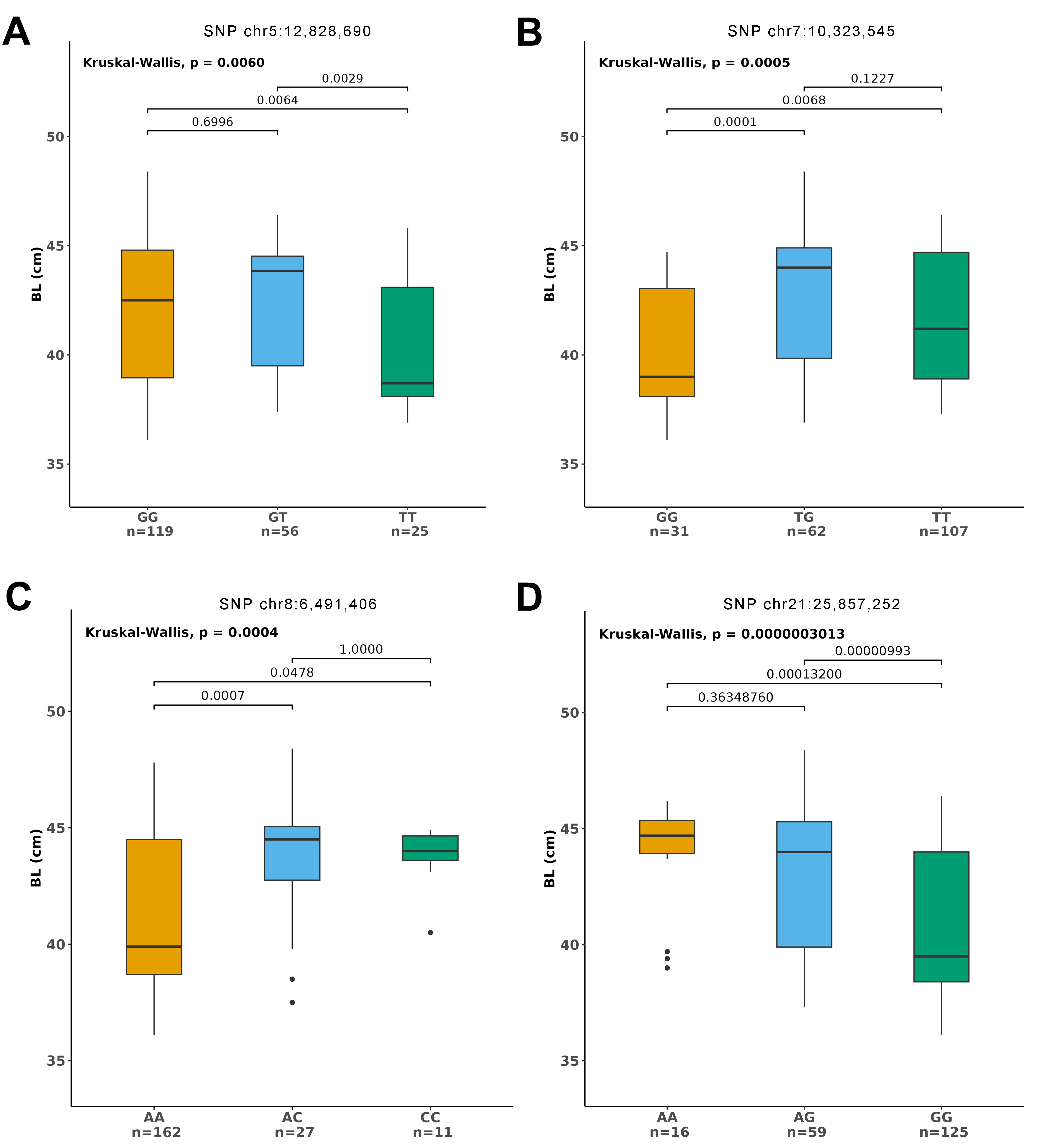

Supplement: Supplementary file 1 [file animals-14-02995-s001.zip › Figure S5.tif]

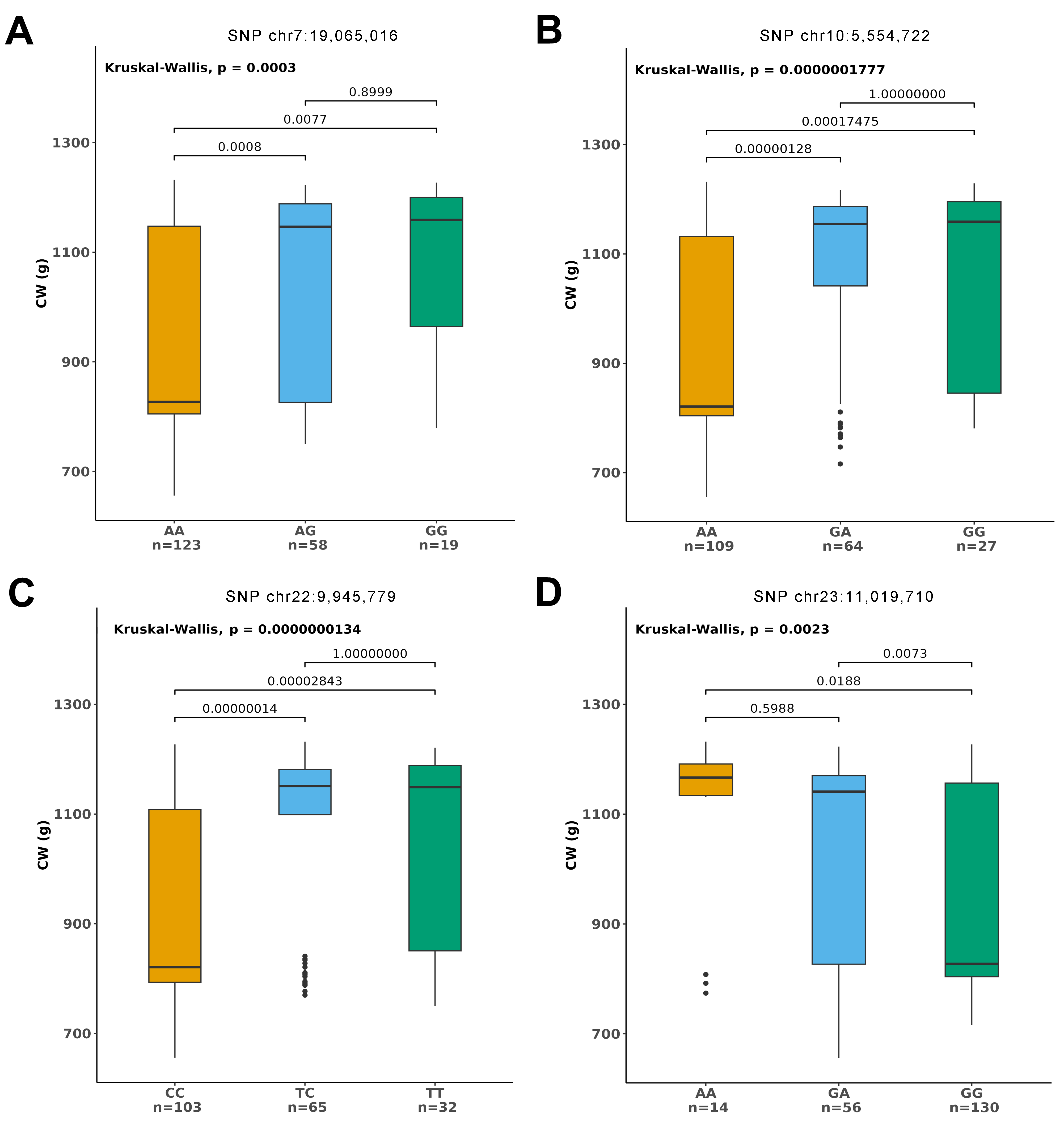

Supplement: Supplementary file 1 [file animals-14-02995-s001.zip › Figure S6.tif]
